# Trends and Transformations: A Bibliometric Analysis of Eye-Tracking Research in Educational Technology

**DOI:** 10.3390/jemr18030023

**Published:** 2025-06-16

**Authors:** Liqi Lai, Baohua Su, Linwei She

**Affiliations:** 1 Modern Education Technology Centre, Jinan University, Zhuhai 519070, China; lailq@jnu.edu.cn; 2 GBA and B&R International Joint Research Center for Smart Logistics, Jinan University, Zhuhai 519070, China; 3 College of Chinese Language and Culture, Jinan University, Guangzhou 510632, China; 4 International Business School, Jinan University, Zhuhai 519070, China

**Keywords:** eye tracking, educational technology, bibliometric analysis, research trends

## Abstract

This study employs bibliometric analysis to provide a comprehensive review of eye-tracking research in the field of educational technology. The study analyzed 374 relevant papers published in 19 high-quality journals from the Web of Science core collection between 2001 and 1 June 2024. The findings reveal research trends, hot topics, and future directions in this field. The findings indicate an upward trend in the application of eye-tracking technology in educational technology, with a significant increase noted after 2014. China, the United States, Germany, and the Netherlands dominate research in this area, contributing to a substantial amount of high-quality research output. Keyword co-occurrence analysis reveals that terms such as “attention,” “cognitive load,” “information,” and “comprehension” are currently hot topics of research. Burst keyword analysis further reveals the evolution of research trends. These trends have shifted from an initial focus on information processing and application studies to a growing emphasis on learner understanding and behavior analysis, ultimately concentrating on learning outcomes and the exploration of emerging technology applications. This study not only provides researchers in the field of educational technology with a comprehensive understanding of the current state of eye-tracking research but also points to future research directions, particularly in optimizing instructional design, enhancing learning outcomes, and exploring the applications of emerging educational technologies using eye-tracking technology.

## 1. Introduction

Educational technology is an interdisciplinary field involving pedagogy, psychology, computer science, communication technology, and information science, among others [1]. It primarily focuses on how to use various technological means to enhance the effectiveness, efficiency, and accessibility of education and learning [2]. The definition of educational technology continues to evolve with technological advancements and societal progress, but the core goal remains to support and enhance the learning process [3,4].

Eye-tracking technology has been used to evaluate and improve digital learning content [5,6]. By analyzing learners’ eye movement behavior, researchers can propose new eye movement metrics to explain this behavior and design more engaging digital learning materials accordingly. Additionally, eye movement pre-training has been found to facilitate the understanding of processes and functions within technical systems. However, the impact of this pre-training on learning outcomes does not directly manifest in learners’ eye movement patterns.

In the field of education, eye tracking review papers cover a multitude of topics, including video learning, special education, mathematics education, multimedia learning, and language learning [5,7,8,9]. However, there are no review papers on eye-tracking research in educational technology. Therefore, conducting research in this area is essential. Based on this foundation, the present study primarily focuses on addressing the following research questions:

RQ 1: What is the overall bibliometric data from the eye-tracking research literature in the field of educational technology?

RQ 2: What are the emerging trends and issues in eye-tracking research in the field of educational technology?

## 2. Literature Review

The author searched the Web of Science database and found that there are currently over 150 review articles on eye-tracking research, indicating that this area has received widespread attention from researchers. The research fields involved include education, healthcare, architecture, finance, automotive, library and information science, and more [8,10,11,12,13,14,15,16,17,18].

In the field of education, eye tracking review papers cover a wide range of topics, including video learning, special education, mathematics education, multimedia learning, and language learning. Below are detailed descriptions of several review papers.

Donmez [8] presented an overview of the utilization of eye-tracking technology within special education. Upon examining 48 relevant studies, it became evident that the integration of eye-tracking technology in special education research is increasingly prevalent. The majority of these studies focused on participants aged between 6 and 11 years. The primary learning outcomes examined in these studies were reading and word learning. Eye-tracking technology has been employed to investigate various aspects of special education, including reading, word learning, mathematical skills, social communication, language development, motor learning, vision improvement, and rote learning. The coding framework of the systematic review encompassed five key categories: Target Disability, Learning Outcome, System Utilized, Eye Tracking Measures, and Participants.

Deng & Gao [7] conducted a systematic review of 44 studies on video learning that utilized eye-tracking technology over the period from 2010 to 2021. Their analysis revealed that not all studies elucidated the mechanisms underlying effective video learning through the application of eye-tracking technology. Furthermore, only a limited number of studies have examined the intricate relationships between eye-tracking indicators and the cognitive activities these indicators reflect. The coding framework of the systematic review encompassed four dimensions: Student Factors, Teaching Context, Learning-Focused Activities, and Learning Outcomes. Learning-focused activities primarily centered on various screen regions, including the instructor area, social cues, content area, non-social cues, progress bar, caption area, blank area of the screen, and the entire screen. Additionally, psychological scales were employed in conjunction with eye-tracking methods to provide supplementary insights. These scales included measures of Cognitive Load, Social Presence, Engagement, and Situational Interest.

Strohmaier et al. [9] reviewed 161 studies on eye tracking in mathematics education published between 1921 and 2018 to assess the fields and themes involved, the methods of use, and the relationship between eye tracking and mathematical thinking and learning. The results showed that most studies focused on the field of numbers and arithmetic, but a significant number of studies in other areas of mathematics education were also researched. The systematic review’s coding of the included literature includes seven categories: Publication, Domain and Topic, Apparatus, Stimuli, Sample and Research Design, Data Treatment, and Interpretation.

Alemdag & Cagiltay [5] analyzed 52 articles to explore the use, methods, and results of eye-tracking technology in multimedia learning research and found that eye-tracking technology can help researchers better understand learners’ learning behaviors and cognitive processes, providing valuable insights for the improvement of teaching and learning strategies. Additionally, eye-tracking technology can be used to assess the usability and effectiveness of multimedia learning materials, offering beneficial suggestions for future research and practical applications.

Based on the author’s comprehensive literature review, no existing review papers on eye-tracking research within the domain of educational technology have been identified. Consequently, conducting research in this area is deemed highly necessary.

## 3. Research Design

### 3.1. Methodology

Bibliometric analysis serves as a quantitative tool for measuring and analyzing pivotal indicators within scholarly publications across specific domains, leveraging extensive databases to generate comprehensive knowledge maps [19]. This analytical approach empowers researchers to aggregate publication data by year, author, institution, journal, and discipline and also facilitates the examination of collaborative networks between authors and institutions. It further supports sophisticated analyses, such as co-citation, co-authorship, and co-word studies. The advent of visualization analysis applications, including CiteSpace, CitNetExplorer, Gephi, Pajek, and VOSviewer [20], has significantly enhanced the capacity for analyzing, constructing, and visualizing bibliometric networks. Utilizing bibliometric techniques, researchers are able to discern clusters of keywords, discern research trends, pinpoint primary research topics, and identify potential future research trajectories within a given field. Ultimately, bibliometric analysis offers an integrative, panoramic perspective derived from the systematic review and synthesis of a substantial corpus of relevant literature.

### 3.2. Bibliometric Analysis Visualization Tools

The present bibliometric analysis utilizes twoprimary visualization tools, Bibliometrix and CiteSpace, all of which are developed using R programming language. Bibliometrix, an open-source tool coded in R, is specifically tailored for comprehensive science mapping analysis [21]. This tool enables researchers to delineate the knowledge base and intellectual structure of a research field or topic, scrutinize research frontiers and conceptual frameworks, and construct social network structures within specific scientific communities. CiteSpace is a visualization tool based on citation data, which reveals the knowledge structure, thematic evolution, and emerging trends in research fields through methods such as co-occurrence networks, cluster analysis, and temporal progression. Its core functionalities include author co-citation analysis, keyword co-occurrence analysis, and document co-citation analysis, making it suitable for systematic literature reviews and scientific mapping [22].

### 3.3. Research Design Process

**Figure 1:**
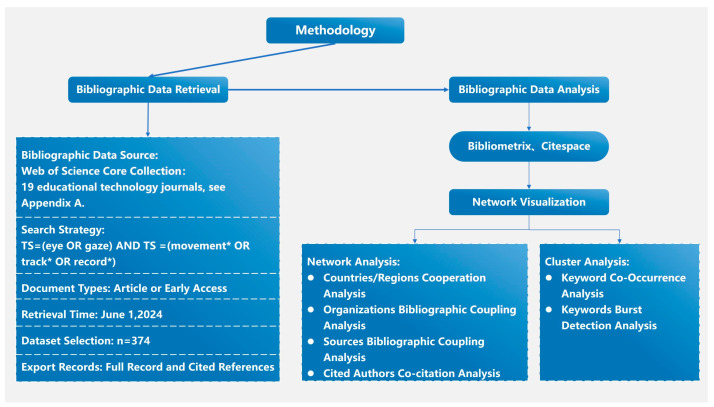
Research design framework.

The research framework of this study is inspired by several previous studies [23,24], and we proceed with the research in steps such as retrieval scheme design, data collection, and data analysis, as shown in Figure 1.

#### 3.3.1. Paper Retrieval Scheme Design

The Web of Science Core Collection, maintained by Clarivate Analytics, serves as the primary source for retrieving papers for analysis. This multi-database platform, known as Web of Science (WOS), encompasses the Science Citation Index Expanded (SCI-Expanded), Social Science Citation Index (SSCI), and Arts & Humanities Citation Index (A&HCI). It features a diverse array of high-impact international journals and is extensively utilized by researchers globally. Through searches conducted within WOS, users can specify a topic and its publication period, subsequently obtaining reliable data via keyword or term co-occurrence analysis [25].

To obtain the analysis data required for this study and ensure the inclusion of high-quality analytical research, the primary database used is Web of Science, an important scientific literature indexing tool [26]. Referring to the methods adopted by Bozkurt [27] and Chang et al. [28], this study selected representative high-quality journals in the field of educational technology. A total of 19 journals were included, with their positions in the JCR quartiles detailed in Appendix A. All these journals fall within the category of “Education & Educational Research,” which is the most commonly researched area in educational technology and is frequently used for academic analysis by scholars [29]. Integrating several previous studies [7,8], the search keywords for this study were formulated as combinations of “eye,” “gaze,” “movement,” “track,” and “record.” The following strategies were employed: (1) The search was restricted to the 19 selected journals using the “SO” field tag, linked together with “OR”; (2) The subject search type was defined as TS = (eye OR gaze) AND (movement* OR track* OR record*), including documents with related keywords; (3) The search expressions from the above two parts were combined using “AND,” with the language limited to English and the document type limited to articles. The search was for “articles” in the English language, and there was no restriction on the publication date. Table 1 displays the specific parameters for the literature search.

#### 3.3.2. Data Collection

All selected papers underwent a screening process, and any paper meeting one or more of the following criteria was excluded (Table 2):

(a) Publications that did not emphasize the topic of this study (i.e., eye tracking);
(b) Articles that were not peer-reviewed;
(c) Works that were not published in English;
(d) Articles that were not accessible through the authors’ institutional database subscription.

#### 3.3.3. Data Processing and Analysis

We conducted the search using the methods mentioned above and retrieved 397 papers. After applying the inclusion criteria, a total of 374 papers were ultimately included. The data was analyzed and synthesized using three analytical methods: (a) descriptive information analysis, (b) keyword co-occurrence analysis, and (c) keyword burst detection analysis.

(1) Descriptive Information Analysis: The descriptive information analysis was conducted using the biblioshiny package to identify the number of publications per year, scientific information sources, prolific authors, and highly cited documents. The general steps for conducting descriptive information statistical analysis with Bibliometrix are as follows: (1) Identify research-related search terms to ensure that subsequent analyses cover all major literature in the knowledge domain. (2) Data collection (titles, authors, keywords, abstracts, etc.): Download bibliometric records required for analysis through databases such as WOS and export them in plain text format. (3) Upload bibliometric records to Bibliometrix. (4) Within Bibliometrix, select the corresponding modules to obtain relevant statistical content. (5) Generate charts for the corresponding statistical content and save them as images or tables.
(2) Keyword Co-occurrence Analysis: A keyword co-occurrence analysis was conducted utilizing CiteSpace 6.3.R1 to pinpoint keywords that frequently co-occur within the retrieved literature [30]. The procedure for performing co-occurrence analysis with CiteSpace is outlined below: (1) Launch CiteSpace and upload the plain text data file from WOS into CiteSpace. (2) Extract keywords from bibliometric records, including both author-provided terms and index-generated terms such as Keywords Plus^®^. (3) Apply time slicing to specify the range of the entire time interval and the length of individual time slices. In this study, the time interval was set from 2001 to 2024, with each time slice representing a 3-year period to capture the evolving trends effectively. (4) Select thresholds and criteria that represent the threshold level for co-occurrence analysis. For this study, the threshold was set to a minimum co-occurrence of 10 times to ensure the identification of significant and frequently discussed keywords. (5) View the visualization map. (6) Interact with the visualization, manipulating the display of visual properties by showing or hiding link strengths and using aliases to merge nodes [31].
(3) Keyword Burst Detection Analysis: Burst keywords were identified using CiteSpace 6.3.R1 and Kleinberg’s burst detection algorithm [31], in conjunction with Gephi, enabling researchers to pinpoint emerging research interests within a specific domain and visualize keywords that exhibited high-frequency occurrences over a defined time interval. Kleinberg [32] proposed a two-state model to represent the non-burst phase (0) and the burst phase (1). For each state, the expected relevant occurrence probability of a specific word is denoted as *p*_0_ or *p*_1_, based on the word’s occurrence during the study period and the total number of documents, following a binomial distribution. Kleinberg constructed a cost model, as shown in Equation (1), where *i* represents the states 0 or 1, p*_i_* denotes the expected relevant occurrence probability of a word under states of 0 or 1, and $\gamma \left(i_{t},i_{t+1}\right)$ is a cost associated with the state transition between 0 and 1. The total cost (TC) of the model is the sum of the negative logarithm of the probability that *r_t_* would generate using the binomial distribution and the transition cost associated with moving from state 0 to state 1. In essence, the burst detection problem is transformed into an optimization problem, aiming to identify the sequence of states for a detection word that yields the lowest total cost output, given the input statistics sequence.

Equation (1): Kleinberg’s [32] cost-model approach to detecting keyword bursts

(1) $$TC\left(i,r_{t},dt\right)=\Sigma \left[-\ln\left(\begin{array}{c} dt\\ r_{t} \end{array}\right)P_{i}^{r_{t}}{\left(1-P_{i}\right)}^{d_{t}-r_{t}}+\gamma \left(i_{t},i_{t+1}\right)\right]$$

## 4. Results

The results of this study encompass the following aspects: (1) Descriptive information analysis, which includes the distribution of publications by year, journal source, and author, as well as the articles with the most citations; (2) keyword co-occurrence analysis; and (3) keyword burst detection to introduce current research topics and future research directions.

### 4.1. Descriptive Bibliometric Analysis

#### 4.1.1. Publication Time

**Figure 2:**
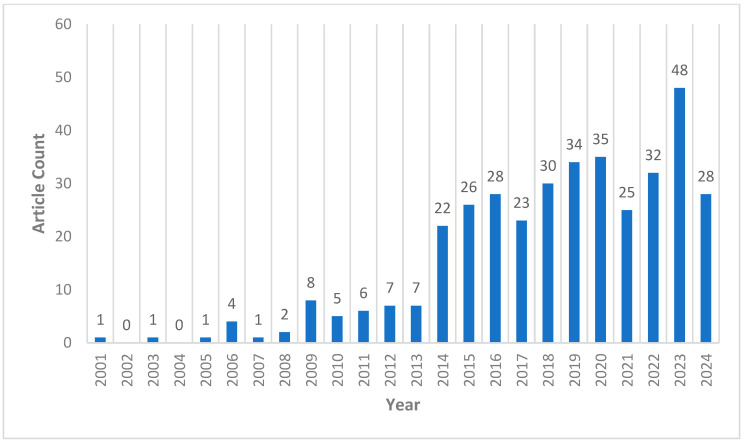
Distribution of publications by year.

Analyzing publication trends across the years can assess activity within a research field. Figure 2 illustrates the distribution of publication years for eye-tracking research articles published in 19 high-level educational technology journals. The data for 2024 is up to 1 June. From Figure 2, it is evident that the earliest paper was published in 2001 [33]. There is a clear upward trend in the number of publications from 2001 to 2024, with a significant increase in the number of papers published after 2014.

#### 4.1.2. Countries/Regions Analysis

**Figure 3:**
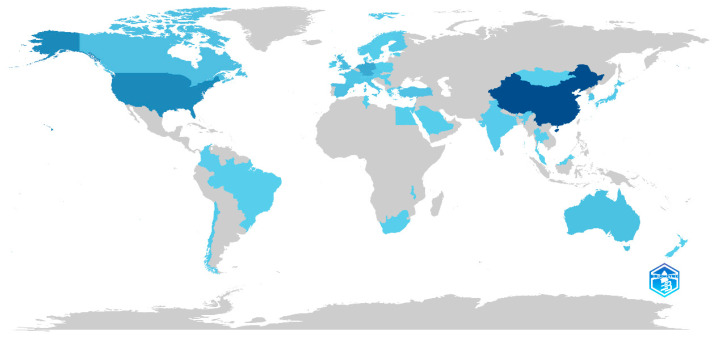
Distribution of publications by country.

**Figure 4:**
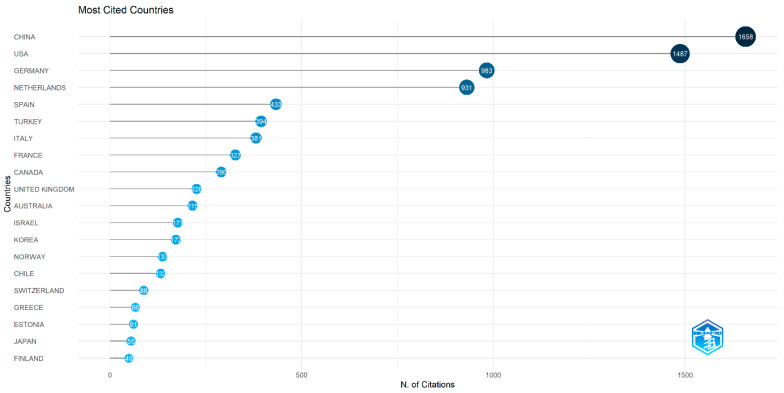
Distribution of the top 20 countries cited.

Figure 3 provides a visual analysis of the distribution of countries where authors of eye-tracking research in the field of educational technology are based. Table 3 lists the top 20 countries by the number of publications, and Figure 4 lists the top 20 countries by the number of citations, both ranked according to the quantity of documents or citations. From Figure 3 and Figure 4 and Table 3, it is evident that China ranks first in both the number of publications and the number of citations, with the USA, Germany, and the Netherlands consistently ranking in the top four for both metrics.

#### 4.1.3. Journal Distribution

**Figure 5:**
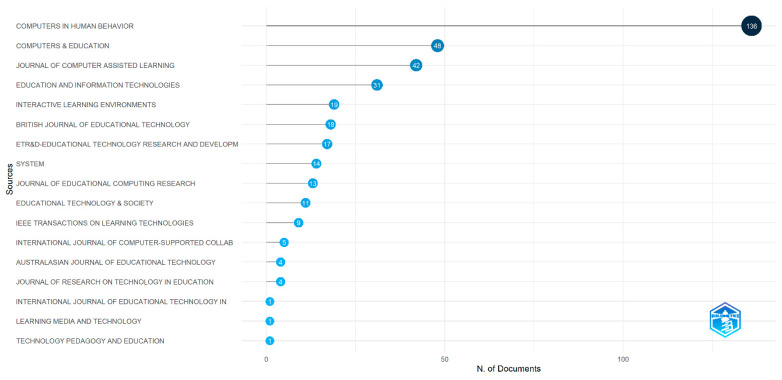
Publications in journals.

This study initially selected 19 core journals in the field of educational technology [24], but after searching the Web of Sciences database, only 17 journals were found to have published papers related to eye-tracking research, with the specific number of publications shown in Figure 5. From Figure 5, it is evident that *Computers in Human Behavior* has a significant lead in the number of publications, being the journal with the highest number of eye-tracking research papers, with 136 articles published. *Computers & Education* and the *Journal of Computer Assisted Learning* follow closely behind, with both journals publishing over 40 papers on eye-tracking research.

#### 4.1.4. Prolific Authors

**Figure 6:**
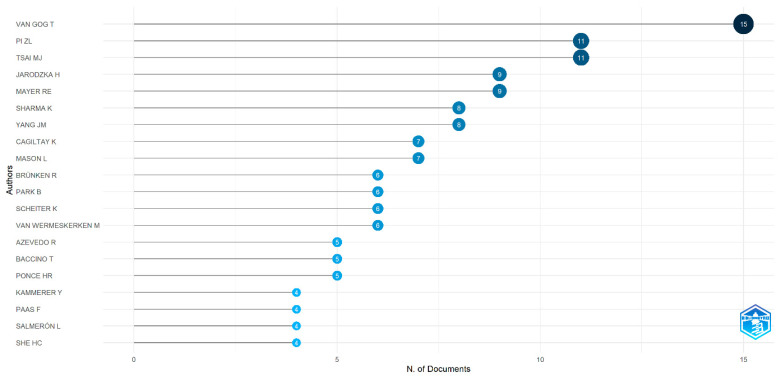
Top 20 published authors.

**Figure 7:**
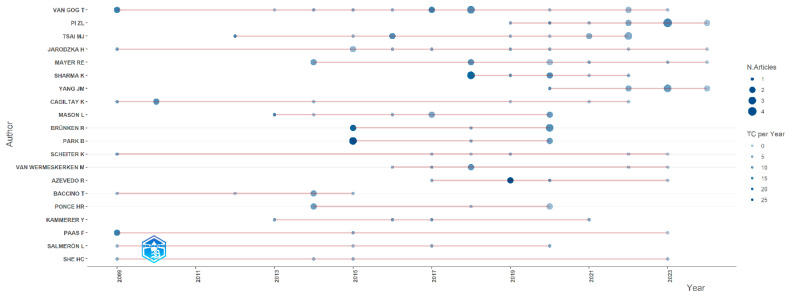
Top 20 published authors’ annual publications.

Figure 6 presents the top 20 most prolific authors, ranked by the number of publications they have produced. The top five authors, along with their publication counts and countries, are VAN GOG T (15 publications), Pi ZL (11 publications/China), TSAI MJ (11 publications), JARODZKA H (9 publications), and MAYE RE (9 publications). Additionally, Figure 7 displays the publication trends of the top 20 authors over different periods, with the red line representing the timeline. The size of the bubbles is proportional to the number of publications per year, and the intensity of the color corresponds to the total number of citations per year. It can be observed from this figure that the prolific authors VAN GOG T and JARODZKA H have been engaged in eye-tracking research for an extended period, starting as early as 2009 and continuing to the present. Notably, these results are based on papers related to eye-tracking research from 19 journals.

#### 4.1.5. Most Cited Documents

Global Citations (GC) measure the impact of a document on the entire database collection, provided by Clarivate. On the other hand, Local Citations (LC) measure the citations of a document within the analyzed set of documents (in this case, *n* = 374 documents). These two indices represent the influence of documents on educational technology eye-tracking research. According to Table 4, based on GC, the most cited document is “Visual attention for solving multiple-choice science problems: An eye tracking analysis” by Tsai et al. [34], followed by Mason et al. [35] and Van Gog et al. [36] in the second and third places, respectively.

### 4.2. Keyword Co-Occurrence Analysis

**Figure 8:**
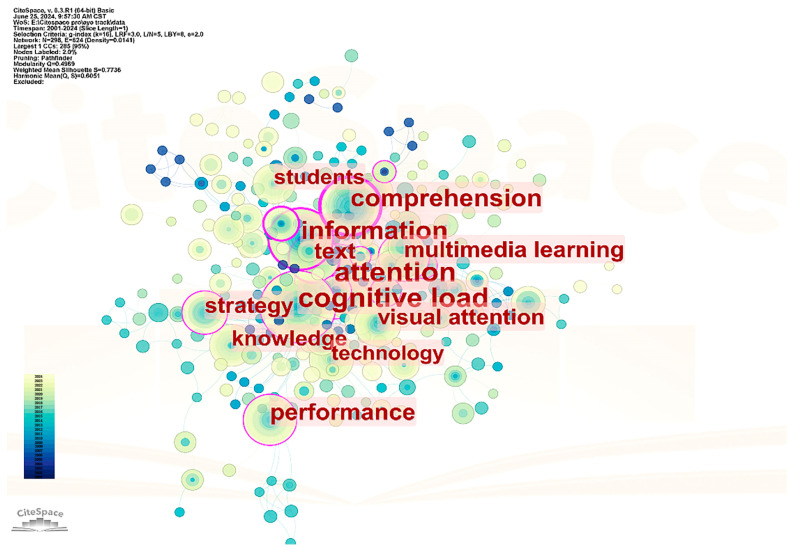
Keyword co-occurrence network with top occurring keywords.

Following the filtering process outlined in the Data Processing and Analysis section, a keyword co-occurrence network was created by identifying clusters of keywords from the 374 selected publications that frequently appeared together and exhibited a high degree of interrelatedness. Based on the established selection criteria, the top 50 keywords for each year were extracted using CiteSpace, represented as nodes interconnected by links. This resulted in a total of 298 keywords and 1631 links. Keywords and links that appeared more than 10 times are depicted and visualized in Figure 8, which features a network map with purple rings. Additional characteristics of the network are detailed in Table 5. Modularity, a key metric ranging from 0 to 1, reflects the overall structural properties of the network and indicates how effectively a network can be identified and segmented into distinct blocks [54]. In this keyword co-occurrence network, the modularity value of 0.4969 is relatively high, suggesting a well-organized network [55]. The degree of nodes refers to the level of correlation a node has with other nodes through links within the network. Generally, a higher node degree indicates a stronger correlation with other nodes and signifies a more prominent role for that node in the overall network [24]. In our network, the average node degree was 4.094, indicating that each node or keyword, on average, co-occurred with approximately 4.094 other keywords.

Figure 8 displays the keyword co-occurrence network, showcasing keywords that have appeared together more than 10 times. The size of each node corresponds to the frequency of co-occurrence for the keywords; larger nodes indicate that the keyword has been co-used more frequently. The color of the links connecting the nodes represents the time frame during which the correlation between two keywords was established: links that are bluer signify older correlations, while redder links indicate more recent correlations.

Table 6 provides the frequency of keywords that appeared more than 10 times in the selected publications. A cluster of keywords—including attention, cognitive load, information, and comprehension—exhibited the highest frequencies (nearly or exceeding 40 times), highlighting the central themes of educational technology eye-tracking research: managing learners’ attention distribution, cognitive load, information processing strategies, and ultimately enhancing comprehension through optimized information presentation and processing. These findings are crucial for advancing multimedia learning design and refining teaching methodologies. In addition to these high-frequency keywords, others such as performance, text, multimedia learning, strategy, and visual attention, also frequently appear in the eye-tracking research literature. These keywords underscore the diverse applications of eye-tracking research in educational technology, encompassing the evaluation of learning outcomes, analysis of text and image roles, development of learning strategies, and investigation of visual information processing. The analysis of these keywords clearly indicates that educational technology eye-tracking research is vital in uncovering learners’ cognitive processes and behavioral patterns within multimedia environments. The results not only enhance the understanding of the learning process but also provide empirical evidence for improving instructional design.

Table 7 illustrates the degree of co-occurrence of individual keywords with others. The keyword “information” had the highest co-occurrence degree of 82, indicating its prominence in recent empirical research within educational technology. The co-occurrence degrees for the keywords attention, cognitive load, and comprehension were 80, 71, and 70, respectively. The degree of co-occurrence serves as an indicator of which keywords are most prominent in the educational technology keyword network, reflecting the prevailing research trends in educational technology eye-tracking studies regarding explicitly examined topics.

A comparison of the keywords in Table 6 and Table 7 reveals that the most frequently occurring keywords highlighted in the keyword co-occurrence network align with those that have the highest co-occurrence degrees. Together, these data sources illuminate significant research issues currently being explored and discussed by scholars. Notably, attention, cognitive load, information, and comprehension all rank in the top four positions in both lists.

### 4.3. Keyword Burst Detection Analysis

To delve deeper into the keyword data of educational technology eye-tracking research, keyword burst detection was utilized. This analysis helps to identify emerging research interests within a specific domain, visualize keywords that occur with high frequency within a specific timeframe, and discern trends in educational technology eye-tracking research. Table 8 presents the results of keyword burst detection. The keywords “information,” “applications in subject areas,” and “children” were the first set to burst, with their respective “bursting periods” being 2003–2014, 2009–2017, and 2009–2020. The early bursting periods of these keywords suggest that researchers initially concentrated on these areas as their primary research interests in the field of educational technology eye-tracking research. We refer to this phase as “Information Processing and Application.” Research during this stage mainly explored how learners process and utilize information in multimedia environments, focusing on the impact of information presentation methods, quantity, and structure on learning outcomes. Researchers also applied eye-tracking technology to specific subject matters, such as science, mathematics, and language, to optimize teaching methods across different disciplines. Additionally, there was a particular emphasis on children’s cognition and behavior in multimedia learning environments, using eye tracking to understand their attention distribution and information processing strategies, aiming to design more child-appropriate educational technologies.

A second set of keywords began to burst from 2012 to 2019; these were “comprehension,” “behavior,” “illustrations,” and “patterns,” with bursting periods from 2012 to 2016, 2015 to 2018, 2017 to 2019, and 2019 to 2020, respectively. We call this phase “Comprehension and Behavioral Analysis.” Research during this phase shifted towards enhancing learners’ understanding of multimedia materials, assessing the impact of material complexity, information presentation, and learners’ prior knowledge on comprehension. Researchers also analyzed learners’ behavioral patterns in multimedia environments using eye-tracking technology, including browsing paths, attention allocation, and responses to different learning tasks. Furthermore, the role of illustrations in multimedia learning was studied, exploring how to design effective combinations of text and images to enhance information transfer and understanding. Finally, by identifying and analyzing different learners’ eye-movement patterns, researchers sought to optimize teaching designs to accommodate various learning styles and needs.

After 2020, educational technology eye-tracking research entered a third phase: “Achievement and Emerging Technology Applications.” The keywords included “achievement,” “students,” “technology,” and “education,” with their bursting periods being 2020–2022, 2021–2024, 2021–2022, and 2022–2024, respectively. Research in this phase concentrated on evaluating the impact of educational technology on learners’ academic achievement and exploring the contributions of different teaching technologies and strategies to academic success through eye tracking. Researchers also paid more attention to students’ overall experience and performance in multimedia learning environments, analyzing students’ eye-tracking data to design more personalized and effective teaching plans. Additionally, the research explored the application of emerging technologies such as virtual reality and augmented reality in education, assessing their impact on learners’ cognition and behavior, aiming to provide data support and a theoretical basis for the development of future educational technologies.

## 5. Discussion

### 5.1. RQ 1: Overall Bibliometric Data of Eye-Tracking Research in the Field of Educational Technology

This study systematically reveals the overall bibliometric data of eye-tracking research in the field of educational technology through bibliometric analysis. The results indicate a steady increase in the application of eye-tracking technology in educational technology since 2001, with a significant rise in the number of research papers published after 2014. This trend suggests that the application of eye-tracking technology in educational technology has garnered growing attention and importance from researchers. This growth may be attributed to the rapid development of educational technology in recent years and the increasing recognition of the potential of eye-tracking technology in the field of education [56]. With the continuous advancement of educational technology, researchers have become increasingly aware that eye-tracking technology can provide deeper insights into learners’ cognitive processes and behavioral patterns, thereby offering robust support for optimizing instructional design and improving learning outcomes [57].

From the perspective of country/region distribution, China, the United States, Germany, and the Netherlands lead the research in this field. These countries have not only produced a substantial number of high-quality research papers, but have also attracted extensive citations and attention. This distribution may be linked to these countries’ overall research capabilities in educational technology, their investment in scientific research, and their openness to emerging technologies [58]. For example, China’s rapid development in educational technology benefits from its vast education market and strong emphasis on educational innovation, coupled with significant government investment in educational technology research, which has provided abundant resources for eye-tracking studies. The United States, as a global leader in educational technology research, maintains its leading position in eye-tracking technology due to its robust scientific foundation and interdisciplinary research capabilities. Germany and the Netherlands, leveraging their profound expertise in educational science and psychology, as well as their proactive exploration of educational technology applications, have emerged as key contributors in this field.

Regarding journal distribution, Computers in Human Behavior, Computers & Education, and the Journal of Computer Assisted Learning are the journals with the highest number of published eye-tracking research papers. These journals hold a significant influence in the field of educational technology and serve as important platforms for showcasing eye-tracking research. Their high impact and broad readership attract a large number of high-quality submissions, thereby advancing the research and application of eye-tracking technology in educational technology.

In terms of author distribution, researchers such as Van Gog T, Pi ZL, and Tsai MJ have published numerous high-quality research papers in this field. Their work has not only promoted the application of eye-tracking technology in educational technology but has also provided valuable references and insights for subsequent studies. These authors’ research is often characterized by high academic value and innovation, laying a solid foundation for the application of eye-tracking technology in education [59].

### 5.2. RQ 2: Emerging Trends and Issues in Eye-Tracking Research in Educational Technology

Through keyword co-occurrence analysis and keyword burst detection analysis, this study reveals the emerging trends and issues in eye-tracking research within the field of educational technology. The results of the keyword co-occurrence analysis show that “attention,” “cognitive load,” “information,” and “comprehension” are the core themes of current research. The frequent occurrence of these keywords reflects researchers’ strong interest in understanding and optimizing learners’ attention allocation, cognitive load, information processing, and knowledge comprehension through eye-tracking technology. The emergence of this research trend is likely related to the continuous pursuit of improving learning outcomes and optimizing instructional design in the field of educational technology. With the increasing prevalence of multimedia learning environments, researchers have become more aware that eye-tracking technology can be used to more precisely measure and analyze learners’ cognitive processes, thereby providing support for the design of more effective instructional materials and methods [5,36].

The keyword burst detection analysis further reveals the evolution of research trends. From 2003 to 2014, research primarily focused on the “Information Processing and Application” phase, concentrating on how learners process and utilize information in multimedia environments and the impact of information presentation methods, quantity, and structure on learning outcomes. This research trend was likely associated with the initial exploration of multimedia learning environments in the field of educational technology at that time [49,59]. Researchers have attempted to use eye-tracking technology to understand the information processing mechanisms of learners in multimedia environments in order to optimize information presentation and improve learning outcomes.

From 2012 to 2019, research entered the “Comprehension and Behavioral Analysis” phase, focusing on how to enhance learners’ understanding of multimedia materials through eye-tracking technology and assessing the impact of material complexity, information presentation, and learners’ prior knowledge on comprehension. This research trend was likely related to the field’s deeper understanding of learners’ cognitive processes. With the continuous development of and improvement in eye-tracking technology, researchers were able to more precisely measure learners’ attention allocation and information processing, thereby providing stronger support for optimizing instructional design and improving learning outcomes [5,60].

After 2020, research entered the “Achievement and Emerging Technology Applications” phase, focusing on the impact of educational technology on learners’ academic achievement and exploring the effects of different teaching technologies and strategies on academic success through eye tracking. This research trend was likely associated with the field’s active exploration of emerging technologies. With the rapid development of emerging technologies such as virtual reality and augmented reality, researchers have attempted to use eye-tracking technology to evaluate the application effects of these emerging technologies in education, thereby promoting innovation and development in educational technology [61,62].

### 5.3. Limitations and Future Research Directions

Despite providing a comprehensive bibliometric analysis of eye-tracking research in the field of educational technology, this study still has some limitations. First, the data source of this study primarily relies on the Web of Science Core Collection, which may limit the comprehensiveness of the research. Important studies that have not been included may exist in other databases. Second, this study mainly focuses on journal articles and gives insufficient consideration to other types of research outputs, such as conference papers and technical reports. This may lead to certain recent or cutting-edge research developments not being adequately represented. Furthermore, this study primarily employs quantitative methods during the analysis process, which limits the in-depth interpretation and qualitative analysis of the research content. This may result in some significant research details and discoveries being overlooked.

In response to the above limitations, future research can be expanded in the following areas:

First, expand the data sources. In addition to the Web of Science Core Collection, other significant academic databases, such as Scopus and IEEE Xplore, should be considered to obtain a more comprehensive set of research data. At the same time, attention should be paid to various types of research outputs, including conference papers, technical reports, and blog articles, to capture the latest research trends and developments.

Second, integrate qualitative analysis methods. Future research could incorporate qualitative analysis methods, such as content analysis and case studies, to provide deeper interpretations and discussions of the research content. This would help reveal specific issues, challenges, and solutions related to the application of eye-tracking technology in the field of educational technology, thereby providing more targeted guidance for practice.

Third, focus on interdisciplinary research. The application of eye-tracking technology in educational technology involves knowledge and methods from multiple disciplines, such as computer science, psychology, and education. Future research could emphasize interdisciplinary collaboration and communication to jointly explore the potential applications and innovations of eye-tracking technology in education.

Fourth, investigate new technologies and methods. With the continuous development of technology, future research can focus on the application of emerging eye-tracking technologies and methods in the field of education. For example, leveraging deep learning and artificial intelligence to enhance the processing and analysis of eye-tracking data, exploring more precise and real-time eye-tracking methods, and investigating how to apply these new technologies and methods in educational practice.

Fifth, discussion on learner diversity. Despite significant progress in the application research of eye-tracking technology in the field of educational technology, the current literature has relatively limited engagement with learner diversity. Learner diversity, including differences in age, cognitive profiles, and cultural backgrounds, is of great significance to both eye-tracking research and educational practice. Moreover, cultural background may also affect learners’ attention allocation and information processing methods, thereby impacting the results of eye-tracking research.

Finally, conduct empirical research. Future research should pay more attention to empirical studies, designing rigorous experiments and surveys to validate the actual effects and impacts of eye-tracking technology in the field of education. This would help provide more reliable and convincing evidence support for educational practice.

## 6. Conclusions

This study provides a comprehensive review of eye-tracking research in the field of educational technology through bibliometric analysis. A total of 374 relevant papers published in 19 high-quality journals from the Web of Science core collection between 2001 and 1 June 2024 were analyzed to uncover research trends, hot topics, and future directions in this field.

The findings indicate that the application of eye-tracking technology in educational technology has been on an upward trend, with a significant increase observed after 2014. China, the United States, Germany, and the Netherlands are identified as the dominant forces in this research area, contributing a substantial amount of high-quality research output. Keyword co-occurrence analysis reveals that terms such as “attention,” “cognitive load,” “information,” and “comprehension” are currently hot topics of research. Additionally, burst keyword analysis further illustrates the evolution of research trends. These trends have shifted from an initial focus on information processing and application studies to a growing emphasis on learner understanding and behavior analysis, and ultimately to concentrating on learning outcomes and the exploration of emerging technology applications.

This study not only offers researchers in the field of educational technology a comprehensive understanding of the current state of eye-tracking research but also points to future research directions. In particular, it highlights the potential of eye-tracking technology in optimizing instructional design, enhancing learning outcomes, and exploring the applications of emerging educational technologies. Future research may further investigate how to better leverage eye-tracking technology to support educational practice and drive continuous development in the field of educational technology.

## Figures and Tables

**Table 1:** Search data parameters for document retrieval.

Parameter	Settings
Topic	TS = (eye OR gaze) AND (movement* OR track* OR record*)
Type	Article
Search Time	1 June 2024
Citation Index	SCI-EXPANDED, SSCI, A&HCI
Category	19 journals, see Appendix A.
Language	English

**Table 2:** Inclusion and exclusion criteria.

Type	Inclusion Criteria
Research content standard	(1) The literature primarily focuses on core topics within the field of educational technology; (2) Only papers written in English are included; (3) Only papers that use eye tracking as the primary data collection tool are considered; (4) The research must describe an experiment involving participants and include a detailed description of the research methods applied, including the experimental setting.
Research quality standard	(1) The included literature must be at least five pages in length, excluding reports and short papers that fall below this page threshold; (2) Full-text papers must be accessible through internet sources; (3) The literature must contain comprehensive information elements, including abstracts, author information, keyword fields, and references; (4) The included literature must have undergone standardization through a double-blind peer review process.

**Table 3:** Top 20 countries in terms of publications.

No.	Country	Freq.
1	China	259
2	USA	134
3	Germany	76
4	Netherlands	68
5	United Kingdom	37
6	Canada	32
7	Spain	31
8	Turkey	27
9	Australia	26
10	Italy	20
11	France	19
12	South Korea	16
13	Finland	14
14	Norway	12
15	Czech Republic	11
16	Japan	11
17	Switzerland	11
18	Chile	10
19	Israel	9
20	India	7

**Table 4:** Most cited documents, local and global.

Document	DOI	Year	LC	GC	LC/GC Ratio (%)
TSAI MJ, 2012, COMPUT EDUC [34]	10.1016/j.compedu.2011.07.012	2012	27	178	15.17
MASON L, 2013, COMPUT EDUC [35]	10.1016/j.compedu.2012.07.011	2013	24	167	14.37
VAN GOG T, 2009, COMPUT HUM BEHAV [36]	10.1016/j.chb.2009.02.007	2009	18	128	14.06
OZCELIK E, 2009, COMPUT EDUC [37]	10.1016/j.compedu.2009.03.002	2009	17	129	13.18
OZCELIK E, 2010, COMPUT HUM BEHAV [38]	10.1016/j.chb.2009.09.001	2010	16	173	9.25
TSAI MJ, 2016, COMPUT EDUC [39]	10.1016/j.compedu.2016.03.011	2016	16	83	19.28
SHE HC, 2009, COMPUT EDUC [40]	10.1016/j.compedu.2009.06.012	2009	14	80	17.50
OUWEHAND K, 2015, EDUC TECHNOL SOC [41]		2015	12	56	21.43
VAN GOG T, 2014, COMPUT EDUC [42]	10.1016/j.compedu.2013.12.004	2014	11	70	15.71
JAMET E, 2014, COMPUT HUM BEHAV [43]	10.1016/j.chb.2013.11.013	2014	11	100	11.00
PARK B, 2015, COMPUT EDUC [44]	10.1016/j.compedu.2015.02.016	2015	11	154	7.14
VAN WERMESKERKEN M, 2017, COMPUT EDUC [45]	10.1016/j.compedu.2017.05.013	2017	11	58	18.97
MASON L, 2016, BRIT J EDUC TECHNOL [46]	10.1111/bjet.12271	2016	10	47	21.28
WANG JH, 2020, COMPUT EDUC [47]	10.1016/j.compedu.2019.103779	2020	10	75	13.33
VAN GOG T, 2009, COMPUT HUM BEHAV-a [48]	10.1016/j.chb.2008.12.021	2009	9	114	7.89
LIU HC, 2011, COMPUT HUM BEHAV [49]	10.1016/j.chb.2011.06.012	2011	9	61	14.75
STULL AT, 2018, COMPUT HUM BEHAV [50]	10.1016/j.chb.2018.07.019	2018	9	65	13.85
STARK L, 2018, COMPUT EDUC [51]	10.1016/j.compedu.2018.02.003	2018	9	50	18.00
SCHNEIDER B, 2013, INT J COMP-SUPP COLL [52]	10.1007/s11412-013-9181-4	2013	8	105	7.62
YANG FY, 2013, COMPUT EDUC [53]	10.1016/j.compedu.2012.10.009	2013	8	66	12.12

**Table 5:** Properties of the keyword co-occurrence network.

Characteristic	Value
Nodes	298
Links	1623
Modularity	0.4969
Average degree of nodes	4.094

**Table 6:** Top frequently occurring keywords.

Keywords	Frequency	Keywords	Frequency
attention	70	memory	19
cognitive load	67	acquisition	15
information	49	behavior	14
comprehension	42	model	13
performance	33	prior knowledge	12
text	29	achievement	12
multimedia learning	29	perception	12
strategy	27	design	12
visual attention	26	virtual reality	11
knowledge	24	multimedia	11
students	22	motivation	11
technology	20	science	11
impact	19	education	10

**Table 7:** Top degree of co-occurrence keywords.

Keywords	Degree	Keywords	Degree
information	82	environments	34
attention	80	students	33
cognitive load	71	model	32
comprehension	70	science	30
multimedia learning	53	motivation	29
knowledge	52	behavior	28
performance	49	perception	28
strategy	48	education	26
visual attention	46	multimedia	24
memory	41	design	23
text	39	applications in subject areas	21
impact	38	cognitive processes	21
prior knowledge	36	patterns	21
acquisition	36	achievement	21
technology	35	seductive details	20

**Table 8:** Keywords burst detection analysis results.

Keywords	Begin	End	Keywords	Begin	End
information	2003	2014	patterns	2019	2020
applications in subject areas	2009	2017	achievement	2020	2022
children	2009	2020	students	2021	2024
comprehension	2012	2016	technology	2021	2022
behavior	2015	2018	education	2022	2024
illustrations	2017	2019			

## Data Availability

The data presented in this study are available on request from the authors.

## Appendix A. Journals Included in This Analysis

**Table:**

No.	Source Title	Category Rank	Category Quartile	Impact Factor (2023)
1	Computers in Human Behavior	3	Q1	9
2	Computers & Education	3	Q1	8.9
3	International Journal of Educational Technology in Higher Education	4	Q1	8.6
4	British Journal of Educational Technology	6	Q1	6.7
5	Internet and Higher Education	8	Q1	6.4
6	Journal of Computer Assisted Learning,	13	Q1	5.1
7	System	15	Q1	4.9
8	Education and Information Technologies	16	Q1	4.8
9	Educational Technology & Society,	22	Q1	4.6
10	Journal of Research on Technology in Education	24	Q1	4.5
11	International Journal of Computer-Supported Collaborative Learning	28	Q1	4.2
12	Journal of Educational Computing Research	33	Q1	4
13	Learning Media and Technology	33	Q1	4
14	Distance Education	45	Q1	3.7
15	Interactive Learning Environments	57	Q1	3.7
16	Technology Pedagogy and Education	60	Q1	3.4
17	ETR&D-Educational Technology Research and Development	64	Q1	3.3
18	Australasian Journal of Educational Technology	64	Q1	3.3
19	IEEE Transactions on Learning Technologies	92	Q1	2.9

Note: The JCR Category of Computers in Human Behavior is Psychology & Experimenta while other journals are categorized as Education & Educational Research.
